# Loss of glutathione peroxidase 3 expression is correlated with epigenetic mechanisms in endometrial adenocarcinoma

**DOI:** 10.1186/1475-2867-10-46

**Published:** 2010-11-24

**Authors:** Eva Falck, Sandra Karlsson, Jessica Carlsson,, Gisela Helenius, Mats Karlsson, Karin Klinga-Levan

**Affiliations:** 1 Systems Biology Research Centre - Tumor biology, School of Life Sciences, University of Skövde, Skövde, Sweden; 2 Department of Pathology, Örebro University Hospital, Örebro, Sweden

## Abstract

Glutathione peroxidase 3 (GPX3) is one of the key enzymes in the cellular defense against oxidative stress and the hepatocyte growth factor receptor, (MET) has been suggested to be influenced by the *GPX3 *gene expression. In a previous microarray study performed by our group, *Gpx3 *was identified as a potential biomarker for rat endometrial adenocarcinoma (EAC), since the expression was highly downregulated in rat EAC tumors. Herein, we have investigated the mRNA expression and *Gpx3 *and *Met *in rat EAC by real time quantitative PCR (qPCR), and the methylation status of *Gpx3*. In addition we have examined the expression of *GPX3 *and *MET *in 30 human EACs of different FIGO grades and 20 benign endometrial tissues. We found that the expression of *GPX3 *was uniformly down regulated in both rat and human EAC, regardless of tumor grade or histopathological subtype, implying that the down-regulation is an early event in EAC. The rate of *Gpx3 *promoter methylation reaches 91%, where biallelic methylation was present in 90% of the methylated tumors. The expression of the *Met *oncogene was slightly upregulated in EACs that showed loss of expression of *Gpx3*, but no tumor suppressor activity of Gpx3/GPX3 was detected. Preliminary results also suggest that the production of H_2_O_2 _is higher in rat endometrial tumors with down-regulated *Gpx3 *expression. A likely consequence of loss of GPX3 protein function would be a higher amount of ROS in the cancer cell environment. Thus, the results suggest important clinical implications of the *GPX3 *expression in EAC, both as a molecular biomarker for EAC and as a potential target for therapeutic interventions.

## Background

Endometrial carcinoma (EC) is the most common gynecological malignancy observed in the western society with an incidence rate of approximately 15-20 per 100.000 women per year. Endometrioid adenocarcinoma (EAC) arises from cells that form the glands in the endometrium. It is the most prevalent subtype constituting approximately 80% of all endometrial cancers. Endometroid andenocarcinoma can be histologically graded according to the FIGO system (International Federation of Gynecology and Obstretics) or classified as low grade or high grade by an alternative architectural binary grading system. The disease may either be an estrogen-dependent low-grade endometroid variant (type I) or a non-estrogen-dependent high grade variant (type II). As expected, estrogen-dependent endometroid carcinomas preferentially affect women in the pre- or peri-menopausal phase, whereas type II EACs usually develops in older post-menopausal women. Type II tumors are typically of high-grade endometroid adenocarcinomas, papillary serous or clear cell types, and generally carry a poor prognosis [1-5].

Due to the complexity of cancer etiology caused by the genetic heterogeneity present in the human population and the influences of environmental factors, it can be advantageous to turn to inbred animal models. In the present and previous works, we have used tumor material from crosses including the BDII/Han rat model, where more than 90% of the female virgins of the BDII inbred rat strain spontaneously develop endometrial cancer during their life time. These tumors are hormone dependent ECs and thus represent spontaneous hormonal carcinogenesis [6-8].

In a recent comprehensive microarray study of endometrial cancer cell lines, we found that the expressions of 354 genes were significantly altered relative to normal/pre-malignant endometrium. When applying traditional statistical analyses and gene classification analysis on the microarray data (Waikato environment of knowledge analysis, Weka), we could identify a three-gene signature (*Gpx3*, *Bgn *and *Tgfb3*), that might have important implications in EAC carcinogenesis [9]. It was also revealed that *Gpx3 *displayed the most significantly altered gene expression in comparisons among endometrial tumors and normal/pre-malignant endometrium.

GPX3 constitutes the backbone of the cellular antioxidant defense system, together with Superoxide Dismutase (SOD) and Catalase (CAT)[10]. GPX3 catalyses the reduction of peroxides and protects cells against oxidative damage. The decreased mRNA expression of *Gpx3 *in rat endometrial tumors might result in an impaired defense against endogenous and exogenous genotoxic compounds, which could potentially lead to an increased mutation rate including genes involved in carcinogenesis. The gene expression of *GPX3 *in human has previously been shown to be silenced in prostate cancer, ovarian clear cell adenocarcinoma, gastric carcinoma and in Barret's disease by epigenetic mechanisms, such as hyper-methylation [11-16]. Furthermore, Yu, et al. [17-23], suggested that GPX3 also contains tumor suppressor activity by, directly or in-directly, regulating cell growth and proliferation through unknown mechanisms. GPX3 may influence the expression of *MET *(mesenchymal-epithelial transition factor) that encodes a tyrosine kinase receptor for hepatocyte growth factor (HGF). Abnormal expression/activation of the MET receptor has been reported in numerous human cancer diseases [17-23].

The aim of this study was to investigate the mechanisms underlying the down-regulation of *Gpx3 *and potential implications in EAC carcinogenesis. The production of hydrogen peroxide in the endometrial tumors displaying loss of *Gpx3 *expression as well as in the endometrial samples with normal/high expression was measured. Since *GPX3 *has been suggested to exhibit tumor suppressor activity, that could regulate the transcription of the oncogene, *Met*, we estimated the correlation of the expression between *Met *and *Gpx3 *by employing a real time RT-PCR expression study. In order to verify the results from the BDII endometrial cancer rat model in human, we also examined the mRNA expression of *GPX3 *and *MET *in human endometroid tumor samples of FIGO grade I, II and III and benign samples.

## Results

### QPCR expression analysis of *Gpx3 *and *Met*

The statistical analyses applied for comparing replicates, revealed no significant differences among the replicates in either of the data sets. An average of the replicates Ct value were used in the following calculations of the relative quantitative gene expression, the delta-delta Ct value.

**Figure 1:**
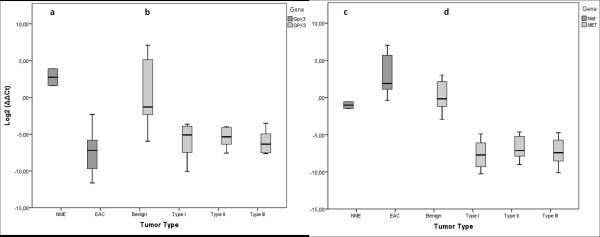
**Gene expression of  of Gpx3/GPX3 and Met/MET in rat  and human.** (a) Gpx3 expression in rat endometrial adenocarcinoma and pre-malignant endometrial cell lines. (b) GPX3 gene expression in human endometrial adenocarcinomas of benign samples and FIGO grade I-III tumor samples. (c) Gene expression of Met in rat endometrial adenocarcinoma and pre-malignant endometrial cell lines. (d) MET gene expression in human endometrial adenocarcinomas of benign samples and FIGO grade I-III tumor samples. The median in each group is represented by a horizontal line that indicates the mean delta delta Ct value in each group.

The majority of the rat tumors displayed an almost total loss of expression of *Gpx3 *whereas the non-/pre-malignant endometrial samples displayed a normal/high expression, as also shown by the highly significant difference among malignant and premalignant/normal samples, (p < 0.001, Figure 1, Table 1). The mRNA expression of *GPX3 *in the human material assessed in 30 human EACs in FIGO grade I-III (10 tumors from each grade), and 21 benign endometrial samples differed significantly between normal and malignant tissues (P < 0.001), but no differences among the different groups of malignant tumors were seen (P > 0.5) (Table 2).

**Table 1:** Overview of the rat material and the tests performed in the study.

Sample	Cell type	Q-PCR *Gpx3*	Q-PCR *Met*	Methylation	Demethylation	H_2_O_2_	FISH
NUT43	NME	X	X	X		X	
NUT56	NME	X	X	X		X	
NUT6	EAC	X	X	X			
NUT12	EAC	X	X	X	X	X	
NUT31	EAC	X	X	X			
NUT39	EAC	X	X	X			
NUT46	EAC	X	X	X			
NUT50	EAC	X	X	X			
NUT51	EAC	X	X	X			
NUT52	EAC	X	X	X			
NUT81	EAC	X	X	X	X		
NUT84	EAC	X	X	X			X
NUT127	EAC	X	X	X			
RUT5	EAC			X			
RUT12	EAC	X	X	X			
RUT13	EAC	X	X	X			
RUT30	EAC	X	X	X			

**Table 2:** Overview of the human material and the tests performed in the study

Sample	Tumor grade	qPCR *GPX3*	qPCR *MET*
Lung	normal	X	X
1-20	benign	X	X
21-30	I	X	X
31-40	II	X	X
41-50	III	X	X

In the rat cell lines, the expression of the *Met *gene was slightly higher among the malignant cell lines (P = 0.054). In the human material the expression of *MET *was significantly lower among the malign tumors (P < 0.001), but there were no differences between FIGO grades (P > 0.5) (Figure 1).

### Epigenetic inactivation of the *Gpx3 *gene

The methylation status of the *Gpx3 *promoter region on bisulfite treated DNA was examined with methylation specific primers (MSP) in methylation-specific PCR of the inbred rat strain BDII, one NME cell line and 15 EAC cell lines (Table 3). The NME cell line was not methylated, while in the BDII rat strain and in 14 out of 15 EAC cell lines (93%), the *Gpx3 *promoter region was hypermethylated of (Table 4).

**Table 3:** Methylation specific primer (MSP) pairs.

Primer pairs	Forward primer	Reverse primer	Fragment size (bp)
Methylated	GTTGTTATTGGTTAGGAAGTTTTCG	GCGTCTTAAAATAACCACCGTC	114
Unmethylated	TTGTTATTGGTTAGGAAGTTTTTGG	AACACATCTTAAAATAACCACCATC	116
*Actb*	GGAAATCGTGCGTGACATTA	AGGAAGGAAGGCTGGAAGAG	183

Methylation specific primer (MSP) pairs for *Gpx3 *used in the methylation specific PCR. The gene *Actb *was used as control.

**Table 4:** Methylation status of the Gpx3 promotor region in the rat EAC cell lines.

		Methylation status	
**Sample**	**Cell type**	**Methylated**	**Unmethylated**
BDII	Strain DNA	M+/M+	
NUT43	NME		M-/M-
NUT56	NME	--------	--------
NUT6	EAC	M+/M+	
NUT12	EAC	M+/M+	
NUT31	EAC	M+/M+	
NUT39	EAC	M+/M-	
NUT46	EAC	M+/M+	
NUT50	EAC	M+/M+	
NUT51	EAC	M+/M+	
NUT52	EAC	M+/M+	
NUT81	EAC	M+/M+	
NUT84	EAC		M-/M-
NUT127	EAC	M+/M+	
RUT5	EAC	M+/M+	
RUT12	EAC	M+/M+	
RUT13	EAC	M+/M+	
RUT30	EAC	M+/M+	

* M+/M+ diallelic methylation, M+/M- monoallelic methylation,

M-/M- unmethylated, ------ no results available

### Demethylation of the Gpx3 gene

**Figure 2:**
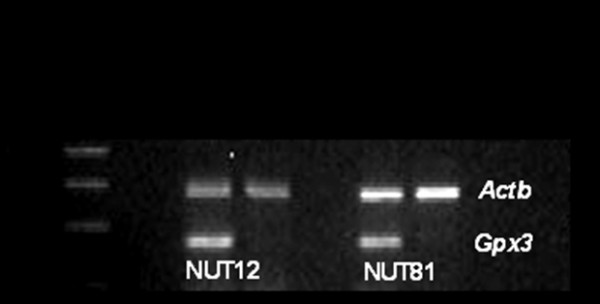
**Demethylation of endometrial tumor cell lines (NUT12 and NUT81) induced activation of *Gpx3 *transcription**. The cells were treated with the demethylating agent 5-aza-2´-deoxycytidine (5Aza-dC) in combination with trichostatin A (TSA), a specific inhibitor of histone deacetylase.

Two of the tumor cell lines (NUT12 and NUT81) that displayed *Gpx3 *promoter biallelic hypermethylation were randomly selected for treatment with the demethylating agent 5-aza-2´-deoxy-cy-tidine (5Aza-dC) in combination with the histone deacetylace inhibitor, trichostatin A (TSA) (Figure 2). As is shown, the *Gpx3 *mRNA expression was fully restored after the demethylation treatment.

### Reactive oxidative species (ROS) in cancer cells

**Figure 3:**
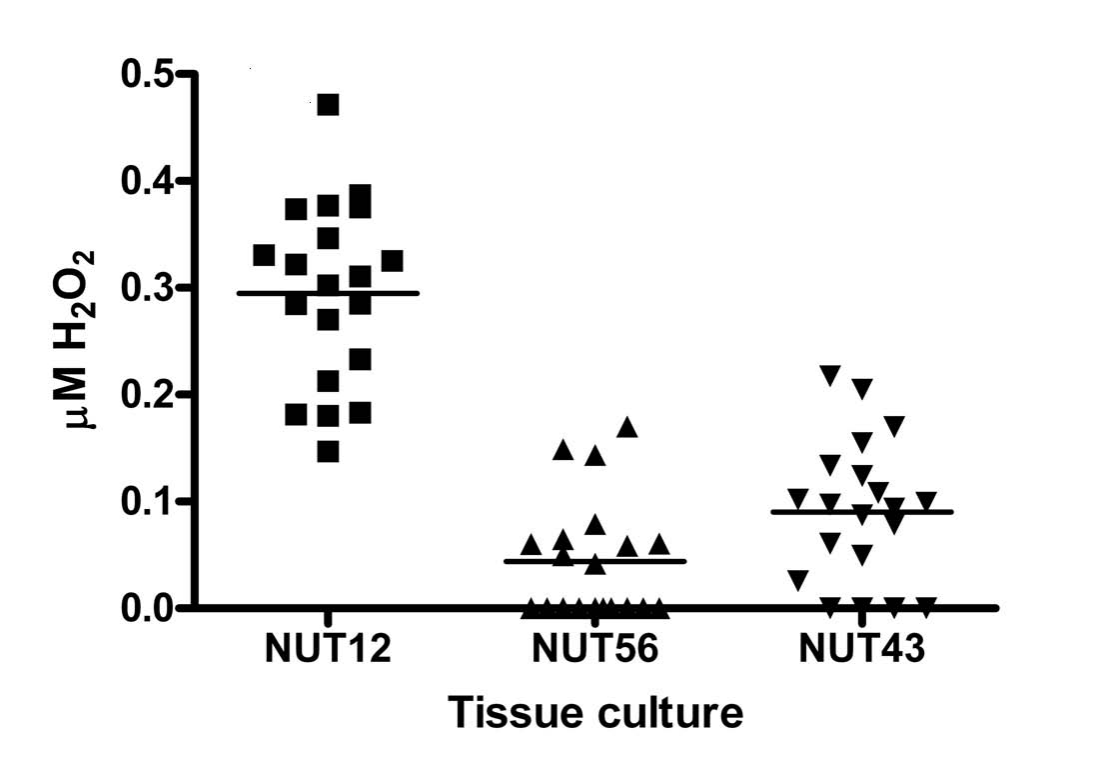
**Measurements of hydrogen peroxide produced in one endometrial tumor with loss of expression of *Gpx3 *and in pre-malignant samples (NUT43 and NUT56) with *Gpx3 *expression**.

The production of hydrogen peroxide in one rat endometrial tumor cell line (NUT12) with loss of expression of *Gpx3 *and in two non-malignant endometrial cell lines (NUT43 and NUT56) with *Gpx3 *expression was measured. A higher generation of hydrogen peroxide was produced in the tumor cell line than in the premalignant cell lines (Figure 3).

### Deletion of the gene Gpx3 in the rat EAC tumor NUT84

FISH was performed on the cell line where *Gpx3 *was unmethylated, but with a decreased gene expression of *Gpx3*. The Gpx3 probe was generated from an NME cell line (NUT43). Six primer pairs representing all exons of *Gpx3 *were designed and used in PCR (Table 5). The PCR products were subsequently pooled, amplified and fluorescently labeled in DOP-PCR. As a positive control, we used a probe generated from a RNO2 BAC. The result of the FISH experiment showed that *Gpx3 *was deleted in the NUT84 cell line.

**Table 5:** Primers used for the construction of the Gpx 3 specific probe.

Primer		Sequence	Product size (bp)
Exon 1	F	TTCTCCCCAAAACCACTGAG	
	R	CCCTTCTCTCCCTCCTAAGC	583
Exon 2	F	GTGGTCCCATGATGCTCTCT	
	R	GGGTTCAAGATTTGGGTGTG	592
Exon 3	F	CTGTAGCAGCCATCCAACTG	
	R	ACCTTGTTCTGTCCGTCACC	588
Exon 4	F	GAAGAGACAGGCTGGGTGAT	
	R	CCCAAAGAGACCACCATCTC	589
Exon 5	F	TCCATGTCAGCCACTCACTC	
	R	GAAGTTGTAGGCCCTGAGA	594
Exon 6	F	TAAGACTGATGCCCCCTCAC	
	R	AGGTTTGAGCAGGACCATTG	587

## Discussion

*Gpx3 *was identified as a potential molecular biomarker for rat EAC in a previous microarray study [9]. In this study, we sought to confirm the previously demonstrated down-regulation of *Gpx3 *expression from the microarray study, of a selection of previously used rat endometrial tumors (Table 1).

As indicated in the previous study, the rat tumors as well as the human tumors indeed displayed an almost total loss of expression of *Gpx3*, whereas the non-/pre-malignant endometrial samples displayed a normal/high expression, p < 0.001 (Figure 1). The mRNA expression of *GPX3 *in the human material was measured in 30 human EACs in FIGO grade I-III (10 tumors from each grade), and 21 benign endometrial samples using qPCR (Table 2). Regardless of tumor grade, the expression of *GPX3 *was low in all tumors, whereas the benign endometrial tissues exhibited a relatively high expression. Thus, in comparisons between tumor FIGO grades we found no significant difference (p > 0.5), while between malignant tumors and benign tissues, we found significant differences, (p < 0.001) (Figure 1). These observations are in agreement with the down-regulated expression of *Gpx3 *in the rat EACs described above. Accordingly, the dramatic loss of *Gpx3 *expression in almost all rat EAC samples and the consistently low expression of *GPX3 *in human EAC, suggest that the down-regulation of *Gpx3 *expression is an early event in EAC carcinogenesis.

In a study performed by Yu *et al*. [17-23], it was suggested that *GPX3 *has tumor suppressor activity as they could show that induced over-expression of *GPX3 *in prostate cancer cell lines decreased invasiveness, anchorage independent growth and colony formation. Moreover, xenografted prostate cancer cells expressing *GPX3*, showed reduction of tumor size, elimination of metastasis and reduction of animal death. Their findings also suggest that the *GPX3 *tumor suppressor activity seems to involve transcriptional regulation of the tumor oncogene, *MET *[12]. MET activation by HGF ligand binding, initiates a whole spectrum of biological activities, including growth promotion, motility and invasiveness [24]. Multiple signaling transduction pathways are induced by MET engagement, including; the MAPK pathway, the RAS pathway, the P13K pathway, the STAT pathway, the beta catenin pathway and the Notch pathway [25]. To investigate whether *Gpx3/GPX3 *regulates the expression of *Met*/*MET *in the rat EAC and human tumor material, we performed qPCR on *Met *as well. The expression of *Met *in the rat tumors was slightly higher in the endometrial tumors with a loss of *Gpx3 *expression. Applying Pearsson´s correlation test, no significant correlation between the *Gpx3 *and *Met *gene expression was detected in the rat tumor material. Thus, in the tumor materials included in this study, we could not confirm any tumor suppressor activity of *Gpx3*. In the human material highly significant differences in the expression of MET between normal and tumor tissues were found (P < 0.001), but not in the way as would be expected from previous studies, where it was suggested that down regulation of *GPX3 *causes up-regulation of *MET *in some cancer types [17-23]. Here, when *GPX3 *was down regulated, also the *MET *gene was down regulated, which was also confirmed by the positive correlation displayed by Pearsson´s correlation test (0.67, P < 0.001). Thus, in this study we could not detect any tumor suppressor activity of *GPX3 *in MET/HGF mediated pathways as suggested by Yu, et al. [17-23].

The GPX3 gene was shown to display epigenetic inactivation in prostate cancer, ovarian clear cell adenocarcinoma, gastric carcinoma and Barret's disease [11-16]. We therefore investigated the methylation status in the BDII inbred rat strain, the EAC cell lines with confirmed decreased mRNA expression (n = 14) in the NME cell lines with a confirmed normal expression of Gpx3 (n = 2) (Figure 1). Hypermethylation of the *Gpx3 *promoter region was found in 14 EAC cell lines. Thirteen of the EAC samples the *Gpx3 *promoter were biallelically methylated (Table 4), One tumor, NUT39, showed monoallelic methylation and *Gpx3 *was therefore not as much down-regulated in this tumor as in the tumors with biallelic hypermethylation, which was also confirmed in the expression analysis (Table 4). The premalignant samples (NME), with an up-regulated expression of *Gpx3*, were not methylated.

Two of the tumor cell lines, NUT12 and NUT81, that displayed biallelic hypermethylation of the *Gpx3 *promoter, were selected for treatment with the demethylating agent 5-aza-2´-deoxy-cy-tidine (5Aza-dC) in combination with the histone deacetylace inhibitor, trichostatin A (TSA). The *Gpx3 *mRNA expression was fully restored after the demethylation treatment (Figure 2). Hence, these results confirm that promoter methylation plays an essential role in silencing of the *Gpx3 *expression.

Cancer cells are constantly exposed to oxidative stress, and it has been shown that human tumor cell lines generate reactive oxidative species (ROS) to a much higher extent than do non-transformed cell lines [26]. When we measured the production of hydrogen peroxide in two rat endometrial tumor cell lines with loss of expression of *Gpx3 *and in one premalignant/normal endometrial cell line with normal *Gpx3 *expression, we found that a higher generation of hydrogen peroxide was produced in the tumor cell lines than in the premalignant cell line (Figure 3). The loss of the protective properties of Gpx3 most likely makes the endometrial cells more vulnerable to ROS damage and genome instability. These findings suggest that the GPX3 function is impaired in endometroid adenocarcinoma, and a likely consequence is an increased amount of hydrogen peroxide and other reactive oxidative species (ROS). Clearly, more tumor cell lines have to be investigated and further functional analyses are required to elucidate the role of ROS in EAC.

In our study *Gpx3 *was strongly down regulated in NUT84. From the result of the methylation studies of this cell line it became clear to us that the loss of expression did not depend on epigenetic inactivation of the gene (Table 4). However, the down-regulation may be explained by other mechanisms, such as structural aberrations at the site of the *Gpx3 *locus. In fact in a previous study a chromosomal deletion has been observed in the region of RNO10 where *Gpx3 *is located, (RNO10q22, 40.3 Mb) which might explain the low expression of the gene [27]. Consequently, we decided to perform FISH on that cell line with a *Gpx3 *probe that was generated from an NME cell line (NUT43) with normal expression of *Gpx3*. From the results of the FISH experiment, we could determine that *Gpx3 *was included in the deletion of that was previously detected [27], which explains the decreased expression of *Gpx3 *in NUT84. As the BDII rat strain did not exhibit a deletion in the region including *Gpx3 *most probably the deletion is an event that has occurred during the tumor development.

## Conclusion

We have found a consistent down-regulated expression of *Gpx3 *in both rat and human EACs. The limited expression of *Gpx3 *in the rat cell lines was correlated to biallelic hyper-methylation of the *Gpx3 *promoter region. Demethylation of the genomes resulted in a restored expression, suggesting that the hypermethylation is responsible for the down-regulation of *Gpx3*. From the FISH images we could confirm that the *Gpx3 *gene was deleted in one of the tumors that was down regulated, but not methylated (NUT84). In previous studies in human prostate cancer, it was suggested that GPX3 exhibits tumor suppressor activity by transcriptional regulation of the oncogene *MET *[11-16]. We could not confirm any such tumor suppressor activity of GPX3/*Gpx*3 either in human or rat endometrial tumors.

It has been proposed that ROS overproduction is required for hypoxic activation of HIF-1 [28] and the results from the preliminary experiments in this study indicate that the GPX3 function is impaired also in endometroid adenocarcinoma. To conclude, the results presented here propose that there are important clinical implications of *GPX3 *expression in EAC, both as an important molecular biomarker for EAC and as a potential target for therapeutic interventions.

## Materials and methods

### Animal crosses and tumor material

The animal material was derived from crosses between BDII/Han females and males from two non-susceptible rat strains, BN/Han and SPRD*Cu3*/Han, where at first an F1 progeny was produced. Subsequently, an F2 offspring by brother/sister mating of the F1 progeny, and a backcross progeny (N1), by crossing the F1 males to BDII females, were produced. The female progeny was palpated twice each week for identification of uterine tumors. Animals suspected to have tumors were euthanized and the tumor tissue surgically removed, subjected to pathological characterization and subsequently used to establishment of cell cultures [29]. In this study we have investigated cell lines established from tissues pathologically classified as endometrial adenocarcinomas (EAC) and from tissues of normal/pre-malignant endometrium (NME). RUT cell lines originate from tumors developed in the F1 and F2 progeny and NUT cell lines originate from the tumors in the backcross progeny (Table 1).

### *In vitro *cell culture conditions

Primary cell cultures established from the EAC tumors were propagated in Dulbecco's modified Eagle medium, supplemented with 100 IU/100 μg/ml penicillin/streptomycin, L-glutamine, MEM amino acids, MEM Non Essential Amino acids, MEM Vitamins solution and 10% heat-inactivated fetal bovine serum, for 3-5 passages in order to obtain the required amount of cells. The NME cell lines were cultured under the same conditions, but in medium containing 20% fetal bovine serum. The cells were grown at 37°C in an atmosphere of 95% humidity and 5% CO_2 _and harvested by trypsinizination at a confluence of 80-90% (approx 1x10^6 ^cells).

### RNA extraction

Total RNA was extracted from the harvested cells of the different endometrial rat cell lines with a KingFisher mL Instrument (Thermo Electron Corporation, USA) according to the manufacturer's protocol (MagAttract Tissue Mini M48 Kit, Qiagen). RNA was spectrophotometrically quantified (NanoDrop technologies, USA).

### Quantitative PCR (qPCR) of *Gpx3 *and *Met *in cell lines from adenocarcinomas (EAC) and normal/premalignant tissue (NME)

A total of 14 EAC and 2 NME cell lines were used in qPCR analysis with *GAPDH *as an endogenous control and Universal Rat Reference RNA, Agilent Technologies, Inc as a calibrator (Table 1). RT-PCR was performed using High Capacity cDNA Reverse Transcription Kit according to the manufacturer's protocol (Applied Bio-systems). Template cDNA was added to TaqMan Universal Master Mix (AB; Applied Biosystems, Foster City, CA, USA) in a 12.5 μl reaction with specific pre-designed probes for the *Gpx3 and Met *(Applied Biosystems). Reactions were performed in duplicates and threshold cycle number was averaged. Relative gene expression quantification was calculated according to the comparative Ct method using *GAPDH *as an endogenous control and Universal Rat reference RNA (Stratagene) as calibrator. The relative quantitative gene expression, were determined as follows: 2-(Ct sample-Ct calibrator), where Ct values of the calibrator and sample are determined by subtracting the Ct value of the target gene from the value of the *GAPDH *gene.

### Bisulfite treatment and methylation-specific PCR

One μg DNA from the susceptible rat strain BDII, 15 EAC and 2 NME cell lines were denatured, sodium bisulfite treated and purified using Epitect Bisulfite Kit according to the manufacturer's protocol (Qiagen) (Table 1). As a positive control, DNA from an endometrial cell line (NUT43) with a normal expression of *Gpx3*, and thus unmethylated was treated with methylase and subsequently treated with sodium bisulfite. The modified tumor/control DNA was used as template for methylation-specific PCR. Methylation specific primers (MSP) were designed using the publicly available MethPrimer program http://www.urogene.org/methprimer/[30]. The bisulphite modification of DNA converts unmethylated cytosines to uracils, whereas methylated cytosines will remain unchanged. Bisulphite treated DNA was amplified with either methylation specific or un-methylation specific primer sets. PCR was carried out in a final 25 μl volume containing 50 ng of template DNA The mixture was heated at 94°C for 1 min and then subjected to 35 cycles of 94°C, 55°C and 72°C and a final extension at 72°C for 7 min. The PCR product was analyzed on a 2% agarose gel with appropriate size marker and the absence or presence of PCR product were detected.

### 5-aza-2´-deoxycytidine and trichostatin A (TSA) treatment in rat endometrial tumor cell lines

Two EAC cell lines (NUT12 and NUT81) with confirmed biallelic methylated promoter status of *Gpx3 *were treated with 5-aza-2´-deoxycytidine and trichostatin A (Sigma). Cells were grown in a medium containing 2.5 μM 5-aza-cytidine for 96 hours, with the medium and drug being replaced every 24 hours and the addition of 300 nM TSA was added for the last 16 hours. After 96 hours, the drugs were removed and total RNA for *Gpx3 *RT-PCR expression analysis was extracted using AllPrep RNA/DNA Mini Kit according to the manufacturer's protocol (Qiagen).

### Hydrogen peroxide measurements

The intra- and extra-cellular amount of hydrogen peroxide in the endometrial cell lines were measured using the Amplex^® ^Red Peroxide/Peroxidase Hydrogen assay kit according to the manufacturer's protocol (Molecular Probes, Invitrogen). The three cell lines investigated (NUT12, NUT43, NUT56) were seeded to a 96 wells plate (Corning) with an initial number of 5000 cells/well (Table 1). In each well, the amount of hydrogen peroxide was measured post 72 hours of incubation, as described above. Each cell line was replicated 20 times. The cells were lysated by adding RIPA buffer (25 mM TRIS-HCl pH 7.6, 150 mM NaCl, 1% deoxycholate, 0, 1% SDS and 1% NP40) followed by incubation on ice for 30 minutes. Intra- and extra-cellular H_2_O_2 _concentrations were assessed by pooling 50 μl of cell lysate with 50 μl of used cell culture media.

### Development of FISH probes for *Gpx3*

DNA from the pre-malignant cell line, NUT43, with a normal expression of *Gpx3 *was used to generate a probe that represented only the *Gpx3 *gene. Six primer pairs, specific for the *Gpx3 *gene, with a product size of approximately 600 base pair each (Table 5), were designed by using the Primer 3 program available on the internet: http://fokker.wi.mit.edu/primer3/input.htm. Amplification was performed by PCR and carried out in a final 25 μl volume containing 100 ng of template DNA. The mixture was heated at 94°C for 1 min and then subjected to 35 cycles of 94°C, 58°C and 72°C and a final extension at 72°C for 7 min. Sizes of the PCR products were determined on a 2% agarose gel with an appropriate size marker. The PCR products were then purified by Mini Elute PCR purification kit (QIAGEN) and the concentration of DNA was measured by NanoDrop (NanoDrop Technologies, USA) amplified product. The amplified sequences were then pooled and fluorescently labeled by dNTP in DOP-PCR and subsequently used as a Gpx3 specific probe in FISH.

One probe, which was used as positive control, was developed from a RNO2 BAC clone (CH230-397A17 from BACPAC Resources Center, Oakland, California). The BAC DNA was amplified by DOP-PCR as follows. The reactions were performed in a final volume of 25 μl and with a BAC DNA concentration of 20 ng/μl. The mixture was heated at 94°C for 1 min and then subjected to 35 cycles of 94°C, 55°C and 72°C and a final extension at 72°C for 7 min. The product was verified on a 2% agarose gel with an appropriate size marker. For FISH, the Nick Translation kit from Abbott molecular was used according to the manufactures protocol.

### Human material

A total of 30 EACs in FIGO grade I-III (10 tumors from each grade embedded in archival formalin fixed paraffin (FFPE) were used in the study. Apart from the endometrial tumors, 21 benign endometrial tissues were collected, and reference material from lung was used in the normalization process (Table 2). All samples were anonymous. A pathologist marked the tumor area in samples in the hematoxylin and eosin slide. Using a Tissue Micro Array-equipment (Pathology Devices), 3-4 cores (∅0.6 mm) of tumor tissue was punched out from the paraffin block. After standard tissue sample deparaffinization using xylene and alcohols, samples were lyzed in a Tris-chloride, EDTA, sodium dodecyl sulfate (SDS) and proteinase K containing buffer. RNA was then extracted and used for the real time qPCR.

### Quantitative PCR (qPCR) of *GPX3 *and *MET *in FIGO grade I-III human EACs

Total RNA was extracted and used for qPCR according to the same procedure as for the rat samples.

### Statistical analysis

For statistical evaluations of Ct values for differences among replicates we applied paired samples t-test and for comparisons of normal and malignant tissues independent sample t-test was applied. (PASW Statistics 18, SPSS Inc, Chicago, USA). In both tests the null hypotheses were assuming no differences between replicates, and no differences between tissue types respectively. The Pearson correlation test was performed to check for correlation between the expression of *Gpx3 *and *Met*. The significance levels were set to P < 0.5 in all statistical tests.

## List of abbreviations

EAC: endometrial adenocarcinoma; NME: normal/pre-malignant endometrium; NUT: backcross, rat uterine tumor; RUT: rat uterine tumor

## Competing interests

The authors declare that they have no competing interests.

## Authors' contributions

SK and EF contributed with all original ideas, designed all studies, performed the data analysis and drafted the manuscript. SK and EF carried out the methylation specific PCR and Q-PCR. SK and JC performed the hydrogen-peroxide assays and the de-methylation study. EF, GH and MK were responsible for the human endometrial study. KKL supervised the project and contributed with ideas and took part in the preparation of the manuscript. All authors have read and approved the final version of the manuscript.
